# A pragmatic comparison of two diabetes education programs in improving type 2 diabetes mellitus outcomes

**DOI:** 10.1186/1756-0500-7-186

**Published:** 2014-03-28

**Authors:** Katherine Dorland, Clare Liddy

**Affiliations:** 1 Department of Family Medicine, University of Ottawa, Ottawa Hospital Riverside Campus, 1967 Riverside Drive, Ottawa, ON K1H 7 W9, Canada; 2 Westend Family Care Clinic Family Health Team, Ottawa, ON, Canada; 3 Bruyère Research Institute, C.T. Lamont Primary Health Care Research Centre, 43 Bruyère St., Ottawa, ON K1N 5C8, Canada

**Keywords:** Type 2 diabetes, Self-management, Education program

## Abstract

**Background:**

Although it is clear that education programs constitute key elements of improved diabetes management, uncertainty exists regarding the optimal method of delivering that education. In addition to the lack of consensus regarding the most appropriate delivery methods for these programs, there is a paucity of research which evaluates these methods in terms of specific clinical outcomes. This pragmatic study compares the effectiveness of two distinct diabetes education programs in improving clinical outcomes in patients with type 2 diabetes mellitus in a primary care setting.

**Results:**

The two diabetes education classes (n = 80 enrolled) retrospectively evaluated were ‘the ABC’s of Diabetes’ (one 2-hour didactic teaching session) and ‘Conversation Maps’ (3 highly interactive weekly classes, 6 hours in total). Eligible participants (n = 32) had their charts reviewed and outcome measures (i.e., glycosylated hemoglobin levels (HbA1c), low density lipoprotein (LDL), systolic blood pressure (SBP), diastolic blood pressure (DBP), and weight) recorded 1 year prior to and 6 months following the class. Pre- and post-class outcome measures were compared. A trend towards lower HbA1c was observed after completion of both classes, with an average reduction of 0.2%, and 0.6% after 6 months in the ‘ABC’s of Diabetes’ class and ‘Conversation Maps’ class respectively. A significant decrease in weight was observed 6 months after the ‘ABC’s of Diabetes’ class (p = 0.028), and in LDL after the ‘Conversation Maps’ class (p = 0.049). Patients with HbA1c ≥ 8% showed a drop of 1.1% in HbA1c 3 months after either class (p = 0.004).

**Conclusions:**

No significant difference in outcomes was found between the two diabetes education classes assessed. There was a trend towards improved glycemic control after both classes, and patients with high HbA1c levels demonstrated statistically significant improvements. This indicates that shorter sessions using didactic teaching methods may be equally effective in producing improvements in diabetes self-management as more intensive course formats.

## Background

In Canada, 2.4 million people, or 6.4% of all women and 7.2% of all men, were living with diabetes in 2009 [1]. Diabetes represents a significant burden to the Canadian healthcare system, with $5.6 billion spent by federal and provincial governments to treat diabetes and its complications in 2005 [2], an amount equivalent to 10% of Canada’s annual health care cost. A large body of research points to both diet [3-5] and exercise [6,7] as crucial aspects of effective diabetes management, as well as a synergistic relationship leading to greater sustained improvements when these lifestyle changes are implemented together [8].

Notably, the majority of the burden of diabetes management falls on patients, as they are ultimately responsible for making the lifestyle modifications central in managing their disease. In addition to diet and physical activity, this can include monitoring blood sugar and adhering to medication recommendations. Several systematic reviews of diabetes education programs present improved outcomes such as better glycemic control [9-16] increased weight loss [10,11,13,15], increased knowledge [10,13], decreased blood pressure [10,15,16], a better cholesterol profile [15,16], improved dietary and exercise habits [13,16], and decreased need for diabetes medication [10].

Although it is clear that education programs constitute key elements of improved diabetes management, there is much uncertainty regarding the optimal method of delivering that education. Some evidence reflects that a multi-disciplinary team education session is better [17], or that longer-term interventions with more follow up have more positive effects [17]. Indeed, one study found HbA1c to decrease with additional contact time between patients and educators, with a 1% decrease for every additional 23.6 h of contact [14]. Studies comparing group and individual education sessions in diabetics demonstrate that they are either equally efficacious at improving glycemic control, blood pressure, and body mass index [10,18] or that group sessions are better at reducing HbA1c than individual education [19,20]. Diabetes self-management education sessions which focus on patient-centered empowerment and more interactive aspects, as opposed to didactic teaching methods, have also been shown to be more effective at improving a range of behavioural outcomes [13,21]. In addition to the lack of consensus regarding the most appropriate delivery methods for these programs, there is a paucity of research which compares these methods in terms of specific clinical outcomes in real life clinical settings. Effort should be made to clarify the most beneficial diabetes education method in eliciting improvements in self-management in a practical primary care setting.

The purpose of this small pragmatic trial was to evaluate and compare the effectiveness of two diabetes education programs: the ‘ABC’s of Diabetes’ and ‘Conversation Maps’ [22] classes, in improving clinically significant outcomes in patients with type 2 diabetes mellitus.

## Methods

### Study design and setting

This was a retrospective observational study. Patients with Type 2 diabetes mellitus at two academic family health team (FHT) sites in Ottawa, Ontario were recruited to voluntary diabetes education classes. Family Health Teams consist of a group of family physicians, as well as many other allied health professionals including nurses, dieticians, and social workers. The focus of these teams is often on chronic disease management and disease prevention. In order to fulfill these mandates FHTs often include focused multidisciplinary teams which target the management or prevention of a specific disease, such as diabetes. The diabetes team at each of the study sites consisted of a dietician, nurse, and pharmacist who followed-up with all the patients with type 2 diabetes at the FHTs.

### Participants

Participants who attended either the ‘ABCs of Diabetes’ class or the ‘Conversation Maps class’ from October 27, 2010 to November 22, 2011 were included in the study. Patients at the clinics were free to choose which program they preferred to attend. Patients who did not have a diagnosis of Type 2 diabetes, as well as participants of the ‘Conversation Maps’ class who did not attend at least two of the three classes, were excluded from the study.

Participants had their charts reviewed and objective outcome measures recorded up to 1 year before the class and up to 6 months after the class.

### Diabetes education interventions

#### The ABC’s of diabetes

This class consisted of a single 2-hour education session for patients with type 2 diabetes. The class was introduced on October 27^th^ 2010, and was run by a dietician, nurse, and pharmacist. It covered information on diabetes, strategies for healthy eating and physical activity, and medications to treat diabetes. The class incorporated some patient participation but was mainly didactic.

#### Conversation maps

This course consisted of 2-hour interactive sessions once a week for three weeks (6 hours total), and incorporated a diabetes conversation map with goals in various areas of lifestyle modification Based on principals of self-efficacy, participants are encouraged to reflect, set goals and problem solve to adopt healthier behaviors related to diabetes. This program was implemented due to high level of interest by staff who had attended a training course. It was introduced on March 7^th^, 2011. The sessions involved group discussions on diabetes, physical activity, and nutrition, as well as a 30 minute fitness component.

### Clinical outcomes

The outcomes assessed in this study were glycosylated hemoglobin levels (HbA1c), low density lipoprotein (LDL), systolic blood pressure (SBP), diastolic blood pressure (DBP), and weight. Outcome measures were assessed from 12 months prior to the class through to 6 months after the completion date of the class using the FHT electronic health record. As indicated in the Canadian diabetes clinical practice guidelines, LDL only needs to be measured every 1-3 years unless treatment is indicated [23]. As such LDL values were not as frequently collected and the ‘Post’ LDL value was calculated using the mean LDL from 1.5 to 12 months after the completion of the class. Patients with HbA1c ≥ 8% were also examined in a specific sub-analysis.

### Statistical analysis

To assess the similarity of the two cohorts with respect to baseline characteristics, a chi-squared (χ2) test was used. Pre- and post-intervention data were compared using a paired, 2-tailed *t*-test, after normality of the data was confirmed with the Shapiro-Wilk test. When normality was not confirmed for one of the data sets (e.g., pre- and post-6 months weight in the ‘Conversation Maps’ class), the Wilcoxon Signed Ranks test was used. An independent, 2-tailed, *t*-test was used to compare the change in outcomes between the ‘ABC’s of Diabetes’ and the ‘Conversation Maps’ class. Again, normality of this data was confirmed using the Shapiro-Wilk test. For one of the data sets (change in HbA1c from pre to post 3 months in the ‘Conversation Maps’ class) normality was not confirmed, therefore the Mann-Whitney U test was used for the comparison. A *p* value <0.05 indicated statistical significance. All statistical analyses were carried out using SPSS 20.0.

The study was approved by the Ottawa Hospital Research Ethics Board prior to its commencement. Written informed consent for participation in the study was obtained from participants. Our research study fully complies with the Helsinki Declaration.

## Results

### Study participation

#### The ‘ABC’s of diabetes’

This class ran 9 times between October 27, 2010 and November 22, 2011 with a total of 39 participants enrolled, and an average of 4.3 participants enrolled per class. Overall, 10 patients (26%) cancelled. 17 participants who were not from Family Health Teams studied were excluded as their outcome measures could not be obtained. Of the 12 remaining participants, one did not have a diagnosis of type 2 diabetes mellitus, leaving a total of 11 patients included in the study from the ‘ABC’s of Diabetes’ class.

#### Conversation maps

**Figure 1:**
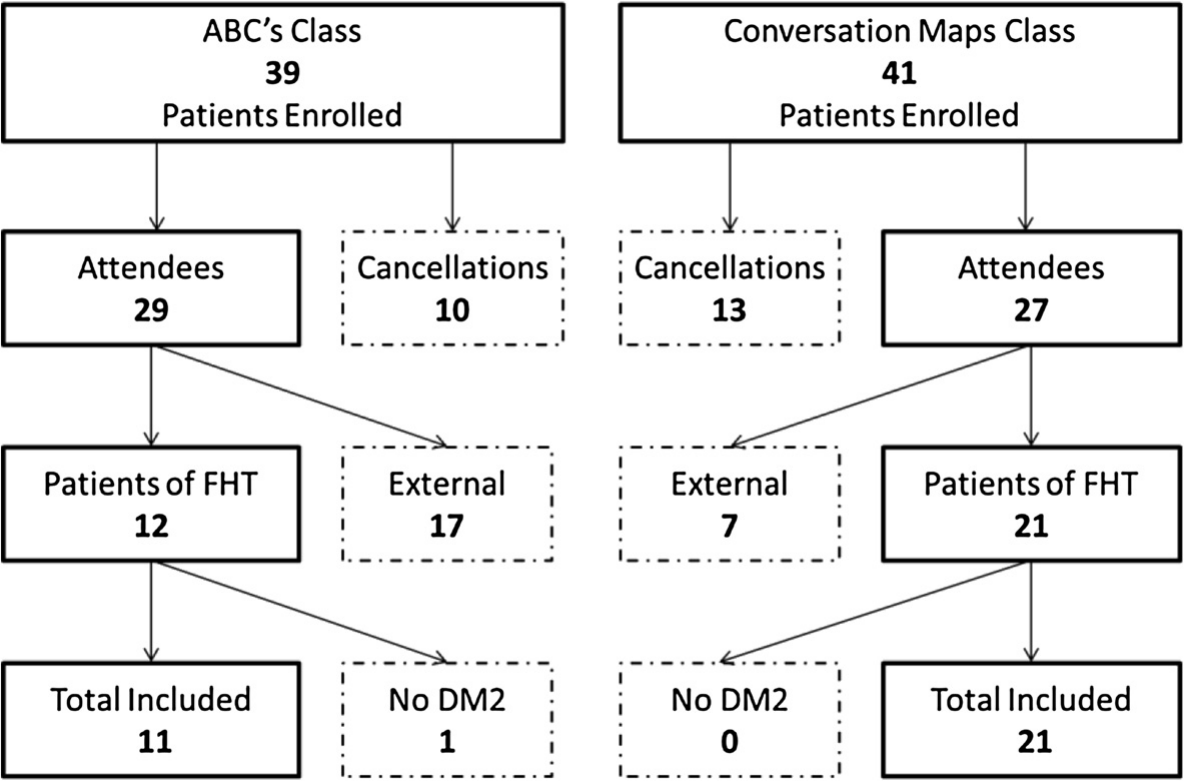
**Flow diagram of patient inclusion.** ABC’s = ‘ABC’s of Diabetes’, FHT = Riverside and Melrose Family Health Teams, DM2 = type 2 diabetes mellitus.

This program ran 6 times between March 7, 2011 and November 7, 2011 with a total of 41 participants enrolled, and an average of 6.8 participants enrolled per class. Overall, 13 patients (32%) cancelled. The 7 participants who were not patients at the Family Health Teams were excluded as their outcome measures could not be obtained. This left a total of 21 patients included in this study from the ‘Conversation Maps’ class. This data is summarized in Figure 1.

The average age of all study participants was 64 years old and 66% (21 out of 32) of them were male (Table 1). There were no statistically significant differences in baseline characteristics of patients in each class type.

**Table 1:** Patient baseline characteristics in each diabetes education class

**Variable**	**Class**		**All**	***p *** **value**
	**ABCs**	**Conversation maps**		
Age (years)				0.429
Mean ± SD	63 ± 11	65 ± 9	64 ± 10	
Range	42-77	48-84	42-84	
Sex (n)				0.540
Male	8	13	21	
Female	3	8	11	
Total (n)	11	21	32	

*p* values calculated using Pearson Chi-Square test, SD indicates standard deviation.

### Effect of ‘ABC’s of diabetes’ and ‘conversation maps’ classes

There was a trend towards lower HbA1c at 3 months and 6 months after the completion of both the ‘ABC’s of Diabetes’ class and ‘Conversation Maps’ classes (Table 2). In the ‘ABC’s of Diabetes’ group there was also a trend towards lower LDL after the class and lower weight at 3 months after the class. Along with the trend towards improvement in HbA1c, the ‘Conversation Maps’ group also showed a trend towards lower SBP and DBP at 6 months post-class, as well as lower weight 3 months after completion of the classes. There was a statistically significant decrease in weight 6 months after the ‘ABC’s of Diabetes’ class (*p* = 0.028), and a significant decrease in LDL after the ‘Conversation Maps’ class (*p* = 0.049).

**Table 2:** Differences in clinical outcomes at 3 and 6 months following “the ABC of diabetes” and “Conversation maps” classes

**Outcome measure**	**‘ABC’s’ of diabetes**			**Conversation maps**			***p *** **value**
	**(Mean ± SD)**			**(Mean ± SD)**			
**Clinical outcomes at 3 months**							
	Pre-Class	3 months	Change	Pre-Class	3 months	Change	
HbA1c (%)	7.8 ± 1.4	7.3 ± 0.7	-0.4 ± 0.8	7.7 ± 1.3	7.4 ± 0.9	-0.3 ± 0.8	0.815
SBP (mmHg)	130.6 ± 9.1	132.6 ± 10.4	2.0 ± 4.7	135.5 ± 8.3	135.3 ± 11.8	-0.2 ± 10.5	0.672
DBP (mmHg)	79.9 ± 7.8	79.9 ± 11.4	0.1 ± 5.8	79.6 ± 7.3	81.4 ± 9.0	1.8 ± 5.6	0.508
Weight (kg)	108.6 ± 54.0	106.6 ± 52.8	-2.0 ± 3.9	96.6 ± 19.3	94.6 ± 20.5	-2.0 ± 8.1	0.999
**Clinical outcomes at 6 months**							
	Pre-Class	6 months	Change	Pre-Class	6 months	Change	
HbA1c (%)	7.1 ± 1.0	6.9 ± 0.9	-0.2 ± 0.3	7.8 ± 1.6	7.2 ± 0.7	-0.6 ± 1.2	0.322
SBP (mmHg)	131.0 ± 10.4	131.7 ± 13.6	-0.7 ± 10.3	134.1 ± 8.5	131.3 ± 7.4	-2.8 ± 12.6	0.546
DBP (mmHg)	79.1 ± 8.7	80.3 ± 9.6	1.2 ± 6.5	80.4 ± 7.2	78.8 ± 7.6	-2.0 ± 7.7	0.373
Weight (kg)	121.1 ± 50.0	118.5 ± 50.2	-2.6 ± 2.4	99.9 ± 42.8	99.1 ± 41.2	-0.8 ± 4.6	0.372
LDL^†^ (mmol/L)	3.0 ± 1.4	2.5 ± 1.1	-0.5 ± 1.2	2.2 ± 0.9	2.0 ± 0.6	-0.3 ± 0.4	0.683

^†^Post-class mean for 1 year following intervention, due to low frequency of readings (as per guidelines).

### Effect of diabetes education classes on outcomes of patients with HbA1c ≥ 8%

The mean HbA1c before the diabetes classes in this sub-group was 9.2% and 3 months after the class the mean had dropped to 8.1%. This drop of 1.1% in HbA1c was statistically significant with a *p* value of 0.004.

### Comparison of the effectiveness of the two diabetes education classes

The change in pre- and post- outcome measures is summarized in Table 2. A comparison of the change in outcome measures between the ‘ABC’s of Diabetes’ and ‘Conversation Maps’ diabetes education classes demonstrated no statistically significant difference between the two classes for any of the outcome measures assessed.

## Discussion

This retrospective observational trial demonstrated a trend towards improved glycemic control for participants in both diabetes education programs, despite differences in class duration and format. These improvements were greater for patients with baseline HbA1c levels ≥ 8%. No difference in outcomes was observed between the two class formats, indicating that shorter sessions using didactic teaching methods may be equally effective in producing improvements in diabetes self-management as more intensive course formats.

The goal of most diabetes treatment methods is to reduce HbA1c. The average decreases in HbA1c in patients who undergo education programs versus control groups vary quite widely in the literature, with a range of 0.26% lower after 4 months [13] to a decrease of 1.4% at 4-6 months [10]. One review article found that if the HbA1c was initially greater than 8%, then self-management education would produce a significant drop in HbA1c [9]. However, in patients with glycosylated hemoglobin levels of less than 7.9% prior to the intervention no significant improvement in glycemic control was observed [9]. The results of our study are in keeping with this pattern. In the ‘ABC’s of Diabetes’ group the average decrease in HbA1c after 3 months was 0.4% and after 6 months was 0.2%. In the ‘Conversation Maps’ group, HbA1c was reduced by 0.3% after 3 months and 0.6% after 6 months. The statistically significant drop in HbA1c of 1.1% observed in our high-risk group (HbA1c > 8) is notable, as this is a highly clinically significant level of reduction, associated with a 21% reduction in death related to diabetes [24].

Although it was hypothesized that the ‘Conversation Maps’ class would be more effective at improving clinical outcomes (as it involves more active patient participation and a longer intervention time) this was not observed. One possible reason that the different class structures elicited the same effect on clinical outcomes was that, due to the small class sizes, the formats may have converged towards a more interactive format. The ‘ABC’s of Diabetes’ class may have been more interactive than expected and both classes were taught by the same instructors, therefore they were likely taught in a similar manner. With an increased sample size, a significant difference may have been seen between the two types of diabetes education programs studied. It is also possible that, though the classes had different structures, they were similar enough to each other to elicit the same effect on clinical outcomes.

This was a pragmatic study and was therefore carried out in an actual clinical setting with diabetes programs already in place and as such there were natural limitations such as selection bias, sample size, and generalizability. Selection bias naturally existed in this retrospective, observational design as participants selected the education program. However, this represents the real life clinical setting where patients are offered a sample of programming and are free to choose what they would like to attend. Supporting patient preference for self-management programs is necessary as the impact on clinical outcomes is dependent on the patient’s ability to modify their lifestyle and how they do that is individualized. Therefore one size does not fit all. The use of pragmatic evaluation tools such as the RE-AIM framework [25] which assess not only the clinical outcomes but also the adoption, implementation, costs and population reach of interventions will assist in understanding the comparative impact of diabetes education programs.

Our sample size was also limited by patient attrition and limited access to clinical data for community-based patients. Many participants in the classes were recruited from the community and were not patients at the academic study-sites, thus their outcome data could not be obtained from the electronic health record. Our analysis therefore may have been underpowered to detect a statistically significant difference in outcome measure improvement between the two education programs. Length of follow-up in our study was limited to 6 months due to logistical issues such as delayed ethics reviews, patient attrition leading to cancelled courses all within the context of a two year training period of the lead investigator (KD). There is a need to study the effect of diabetes education programs over a longer term period. The use of routinely collected clinical data housed in larger health administrative databases would enable both more complete access to clinical and utilization outcomes as well as longer term follow-up (>6 months). The study was carried out in an urban setting, within an academic teaching site in Canada, and may not be widely generalizable to different settings.

Nonetheless, our results represent an important contribution to the knowledge translation literature in terms of bridging the divide between large studies carried out in controlled settings and the reality of implementing evidence based clinical programming.

## Conclusion

With the increasing numbers of people affected by diabetes, it is important to promote patient education and self-management of diabetes. The results of this study comparing the outcomes of two diabetes education programs offered in a primary care practice setting, demonstrate that shorter sessions using didactic teaching methods may be equally effective in producing improvements as more intensive course formats. Targeting those patients with poorer glycemic control produces greater effect.

## Competing interests

The authors declare that they have no competing interests.

## Authors’ contributions

KD and CL have made substantial contributions to conception, design, data analysis and interpretation of data; have been involved in drafting the manuscript; and have given final approval of the version to be published.
